# Annexin A1 Induces Skeletal Muscle Cell Migration Acting through Formyl Peptide Receptors

**DOI:** 10.1371/journal.pone.0048246

**Published:** 2012-10-29

**Authors:** Valentina Bizzarro, Raffaella Belvedere, Fabrizio Dal Piaz, Luca Parente, Antonello Petrella

**Affiliations:** Department of Pharmaceutical and Biomedical Sciences, University of Salerno, Salerno, Italy; University of Birmingham, United Kingdom

## Abstract

Annexin A1 (ANXA1, lipocortin-1) is a glucocorticoid-regulated 37-kDa protein, so called since its main property is to bind (i.e. to annex) to cellular membranes in a Ca^2+^-dependent manner. Although ANXA1 has predominantly been studied in the context of immune responses and cancer, the protein can affect a larger variety of biological phenomena, including cell proliferation and migration. Our previous results show that endogenous ANXA1 positively modulates myoblast cell differentiation by promoting migration of satellite cells and, consequently, skeletal muscle differentiation. In this work, we have evaluated the hypothesis that ANXA1 is able to exert effects on myoblast cell migration acting through formyl peptide receptors (FPRs) following changes in its subcellular localization as in other cell types and tissues. The analysis of the subcellular localization of ANXA1 in C2C12 myoblasts during myogenic differentiation showed an interesting increase of extracellular ANXA1 starting from the initial phases of skeletal muscle cell differentiation. The investigation of intracellular Ca^2+^ perturbation following exogenous administration of the ANXA1 N-terminal derived peptide Ac2-26 established the engagement of the FPRs which expression in C2C12 cells was assessed by qualitative PCR. Wound healing assay experiments showed that Ac2-26 peptide is able to increase migration of C2C12 skeletal muscle cells and to induce cell surface translocation and secretion of ANXA1. Our results suggest a role for ANXA1 as a highly versatile component in the signaling chains triggered by the proper calcium perturbation that takes place during active migration and differentiation or membrane repair since the protein is strongly redistributed onto the plasma membranes after an rapid increase of intracellular levels of Ca^2+^. These properties indicate that ANXA1 may be involved in a novel repair mechanism for skeletal muscle and may have therapeutic implications with respect to the development of ANXA1 mimetics.

## Introduction

Under normal biological conditions adult skeletal muscle is an extremely stable tissue. However, upon damage due to specific diseases, trauma or strong physical exercise, skeletal muscle, as well as myocardium muscle [1], exhibits a remarkable capacity of self-repair aimed at preventing the loss of muscle mass.

Regeneration of skeletal muscle is mainly carried out by satellite cells (SCs) an adult stem cell population associated with myofibers and localized within the basal lamina of the muscle fibers [2]. These resident stem cells are a heterogeneous population composed of stem cells and committed progenitors.

The conversion of activated SCs and myoblasts into terminally differentiated skeletal fibers is a highly regulated process characterized by the sequential induction of muscle specific gene products. Two distinct phases have been reported to be involved in the development and regeneration of skeletal muscle: the SC commitment phase, which requires the activity of primary myogenic factors, MyoD and Myf5, for the propagation and survival of myoblasts, and the differentiation phase, regulated by the expression of secondary myogenic factors, myogenin and MRF4 [3]. This latter stage can be divided temporally into a series of steps including migration, myoblast-myoblast alignment and adhesion, plasma membrane breakdown and the fusion of the cells with damaged muscle fibers or with themselves, to produce new fibers that replace the dead ones [4].

Different factors can modulate SC activity including migration, chemotaxis, proliferation, and differentiation [3].

While a large and detailed body of literature is available in the context of other cell types, particularly neural crest cells, neurons, and endothelial cells, information on SC motility or migration is comparatively scarce, probably due to technical difficulties in visualizing SCs dynamically within the muscle tissue [5]. Due to this limited availability and the restricted number of experimental approaches that can be employed to investigate their biological features *in vivo*, myoblastic cell lines, such as the C2C12 which is derived from mouse muscle SCs, are widely utilized to study *in vitro* skeletal muscle growth and differentiation.

Annexin A1 (ANXA1, lipocortin-1) is the first characterized member of the annexin superfamily of proteins, so called since their main property is to bind (i.e., to annex) to cellular membranes in a Ca^2+^-dependent manner. Originally described as an endogen mediator of the anti-inflammatory effects of glucocorticoids, in the last 20 years ANXA1 has been involved in a broad range of molecular and cellular processes, including acute [6] and chronic [7] inflammation, leukocyte migration [8]–[9], kinase activities in signal transduction [10], preservation of cytoskeleton and extracellular matrix integrity [11], tissue maintenance and apoptosis [6], [12]–[13], cell growth and differentiation [14].

ANXA1 has been shown to localize to the cell surface of various cell types where it is thought to be important in biological function [15]–[20].

It has been shown that regulatory action on cell surface by extracellular ANXA1 is mediated by signaling through FPRs [21]–[23].

FPRs are G-protein coupled chemoattractant receptors, which can sense gradients of bacterial peptides such as Formyl-Methionine-Leucine-Phenylalanine (fMLP) and thereby direct leukocytes towards sites of bacterial infection [24]. Ligand binding to FPR activates a number of downstream effector enzymes including phospholipase C, catalyzing the cleavage of phosphatidylinositol 4,5-biphosphate into secondary messengers inositol 1,4,5-triphosphate and diacylglycerol leading to calcium mobilization and activation of protein kinase C [25]. Although FPRs are classically thought to act as chemotactic receptors regulating leukocyte migration, they have been shown to be expressed in diverse cellular populations and to elicit differential biological responses [26].

Our previous studies [27] indicate that the inhibition of ANXA1 expression by siRNAs in C2C12 cells caused reduction in myogenic differentiation, whereas analysis on sorted quiescent and activated SCs of *Tg:Pax7nGFP* mice showed that ANXA1 is expressed in both quiescent and activated SCs cells. Interestingly, we have shown that ANXA1 expression is not restricted to dividing transit amplifying myoblasts that are generated from SCs after injury, but is present also in the quiescent SCs isolated directly from homeostatic tissue. Immunofluorescence approaches on sections of *Tibialis Anterior* muscle confirmed that ANXA1 is expressed in quiescent and activated SCs co-stained with Pax7 (a marker of SC quiescence) and suggested that the protein is mainly localized in the cells that migrate in the lumen of regenerating fibers.

Moreover, confocal microscopy experiments on C2C12 cell line have shown that ANXA1 is found diffusely through the cytoplasm, although it has an actin-like filamentous organization and is enriched at the lamellipodial extrusions of migrating cells. Finally, ANXA1 neutralizing antibody is able to induce a significant reduction of myogenic differentiation and myoblast cell migration [27].

In the present study we show that ANXA1 can promote skeletal muscle cell migration by acting through FPR receptors possibly leading to fully differentiated cells: *in vivo*, the ultimate outcome would be tissue repair.

## Materials and Methods

### Cell Culture

C2C12, mouse myoblast cells (ATCC, Rockville, MD, USA) were cultured in Dulbecco’s Modified Eagle’s Medium (DMEM; Lonza) containing L-Glutamine 2 mM supplemented with antibiotics (10000 U/ml penicillin and 10 mg/ml streptomycin; Lonza) and containing 10% heat-inactivated fetal bovine serum (FBS; Lonza), referred to as growth medium (GM). To induce differentiation, after reaching cells 80% confluency, GM was replaced with DMEM containing antibiotics and 2% heat-inactivated horse serum, referred to as differentiation medium (DM). Cultures destined for immunohistochemistry were grown to dense confluency on glass coverslips. After reaching 80% confluency, cells were incubated first in Na^+^-Tyrode’s solution (140 mM NaCl, 5 mM KCl, 1 mM MgCl2, 10 mM glucose and 10 mM HEPES; pH 7.4) containing or not 20 µM Ionomycin (Sigma-Aldrich) as a Ca^2+^ ionophore and either 2 mM CaCl_2_ or 2 mM EDTA for 10 min at 37°C.

### Confocal Microscopy

After the specific time of incubation, C2C12 cells were fixed in p-formaldehyde (4% v/v in PBS; Sigma-Aldrich) for 5 minutes. The cells were permeabilized in Triton X-100 (0.5% v/v in PBS) for 5 minutes, and then incubated in goat serum (20% v/v PBS) for 30 minutes, and with a rabbit anti-ANXA1 antibody in PBS (1∶100; Invitrogen) overnight at 4°C. After two washing steps with PBS, cells were incubated with AlexaFluor anti-rabbit (1∶1000; Molecular Probes) for 2 h, and FITC-conjugated Phalloidin (Sigma-Aldrich) for 30 minutes. The coverslips were mounted in glycerol (40% v/v PBS). A Zeiss LSM 710 Laser Scanning Microscope (Carl Zeiss MicroImaging GmbH Jena Germany) was used for data acquisition. To detect nucleus and filaments, samples were excited with a 458 and 488 nm Argon laser respectively. A 555 nm He-Ne laser was used to detect emission signals from ANXA1 stain. Samples were vertically scanned from the bottom of the coverslip with a total depth of 5 µm and a 63X (1.40 NA) Plan-Apochromat oil-immersion objective. A total of 10 z-line scans with a step distance of 0.5 µm were collected and single planes or maximum intensity projections were generated with Zeiss ZEN Confocal Software (Carl Zeiss MicroImaging GmbH Jena Germany).

### Western Blot Analysis

Details of the procedure for immunoblotting have been previously described [12]. After three washing in TBST, the blots were incubated overnight at 4°C with primary polyclonal antibody against ANXA1 (1∶10000; Invitrogen), with primary monoclonal antibodies against MyoD (1∶500; Dako), Myogenin (1∶500; Santa Cruz Biotechnology) and MyHC (1∶500; Santa Cruz Biotechnology) and α-Tubulin (1∶2000; Sigma-Aldrich) and then at RT with an appropriate secondary rabbit or mouse antibody (1∶5000; Sigma-Aldrich). Immunoreactive protein bands were detected by chemioluminescence using enhanced chemioluminescence reagents (ECL; Amersham) and exposed to Hyperfilm. The blots were scanned and analysed (Gel-Doc 2000, BIO-RAD). All results are mean ± SEM of 3 or more experiments performed in triplicate. The optical density of the protein bands detected by Western blotting was normalized on tubulin levels. Statistical comparison between groups were made using Bonferroni parametric test. Differences were considered significant if p<0.01.

### PCR

C2C12 cells were seeded at an initial density of 1×10^6^ in a 100 mm Petri dish and incubated for 48 h in GM allowing cells to reach 90% confluency. Total RNA was extracted from C2C12 cells using Trizol (Invitrogen), according to the manufacturer’s instructions. Total RNA (1 µg) was used to synthesize cDNA using a reverse transcription kit (Promega). PCR was conducted by using the following primers:

Fpr-rs 1 primer pair 1: (fwd 5′-CAG CCT GTA CTT TCG ACT TCT CC-3′) e (rev 3′-ATT GGT GCC TGT ATC ACT GGT CT-5′);

Fpr-rs2 primer pair 1: (fwd 5′-CTT TAT CTG CTG GTT TCC CTT TC-3′) and (rev 3′-CTG GTG CTT GAA TCA CTG GTT TG-5′);

Fpr-rs 1 primer pair 2: (fwd 5′- TCC ATT GTT GCC ATT TGC A -3′) and (rev 3′- GCT GTT GAA GAA AGC CAA GG -5′);

Fpr-rs 2 primer pair 2: (fwd 5′- ACT GTG AGC CTG GCT AGG AA -3′) and (rev 3′- CAT CAG TTT GAG CCC AGG AT -5′)

The predicted Fpr-rs1 primer pairs 1 and 2 and Fpr-rs2 primer pairs 1 and 2 products are 240 bp and 297 bp respectively. The Fpr-rs1 and Fpr-rs2 genes were amplified using PCR under the following conditions: pre-denaturation at 94°C for 2 min, 35 cycles of denaturation at 94°C for 30 s, annealing at 60°C for 30 s, extension at 72°C for 30 s and a final extension at 72°C for 10 min. The products were stored at 4°C. A portion (5 µl) of the PCR product was electrophoresed on a 1% agarose gel in a Tris-acetate-EDTA buffer. The gel was stained with ethidium bromide and was scanned and analysed (Gel-Doc 2000, BIO-RAD).

### Measurement of Intracellular Ca^2+^ Signaling

Intracellular Ca^2+^ concentrations [Ca^2+^] were measured using the fluorescent indicator dye Fura 2-AM (Sigma-Aldrich), the membrane-permeant acetoxymethyl ester form of Fura 2, as previously described [28] with minor revisions.

Briefly, C2C12 cells (1×10^5/^ml) were washed in phosphate buffered saline (PBS), resuspended in 1 ml of Hank’s balanced salt solution (HBSS) containing 5 mM Fura 2-AM and incubated for 45 min at 37°C. After the incubation period, cells were washed with the same buffer to remove excess of Fura 2-AM and then incubated in 1 ml of buffer containing or not 0.1 mM Ca^2+^.

C2C12 cells were then transferred to the spectrofluorimeter (Perkin-Elmer LS-55). Treatments with ionomycin (1 mM) and/or fMLP (50 nM; Sigma-Aldrich), ANXA1 N-terminal peptide Ac2-26 (100 nM; Tocris Biosciences), cyclosporine H (CsH; 500 nM; Alexis-Biochemicals) were carried out by adding the appropriate concentrations of each substance into the cuvette in Ca^2+^ -free HBSS/0.5 mM EDTA buffer.

The excitation wavelength was alternated between 340 and 380 nm, and emission fluorescence was recorded at 515 nm. The fluorescence ratio was calculated as F340/F380 nm.

Maximum and minimum [Ca^2+^] were determined at the end of each experimental protocol by adding to the cells HBSS containing 1 mM ionomycin and 15 mM EDTA, respectively, according to the equation of Grynkiewicz [29].

### In vitro Wound-healing Assay

Details of the procedure for wound healing assay have been previously described [27]. Briefly, C2C12 cells were seeded in a 12-well plastic plate at 2×10^5^ cells per well. After 24 h incubation, cells reached 100% confluency and a wound was produced at the centre of the monolayer by gently scraping the cells with a sterile plastic p200 pipette tip. After removing incubation medium and washing with PBS, cell cultures were incubated in the presence of fMLP (50 nM), Ac2-26 (100 nm), CsH (500 nM) or in GM as control. The wounded cell cultures were then incubated at 37°C in a humidified and equilibrated (5% v/v CO_2_) incubation chamber of an Integrated Live Cell Workstation Leica AF-6000 LX. A 10x phase contrast objective was used to record cell movements with a frequency of acquisition of 10 minutes. The migration rate of individual cells was determined by measuring the distances covered from the initial time to the selected time-points (bar of distance tool, Leica ASF software). For each condition five independent experiments were performed. For each wound five different positions were registered, and for each position ten different cells were randomly selected to measure the migration distances. Statistical analysis were performed by using the Microsoft Excel™ software. Data were analyzed using unpaired, two-tailed t-test comparing two variables. Data are presented as means ± SD. Values <0.01 were considered as significant.

### Proteomic Experiments

The ANXA1 N-terminal peptide Ac2-26 was modified with NH2-PEG4-Biotin (Pierce) leading to the formations of a biotinylated form of the peptide. The derivatization reaction was carried out for 3 hours at room temperature under stirring, incubating 100 µl of a Ac2-26 solution 1 mg/ml acetonitrile 20% with a 10 fold molar excess of NHS-PEG4-Biotin (Pierce). The kinetic reaction was monitored by LC-MS, using a Q-TOF Premier instrument (Waters). Even if in the peptide sequences are present two Lys residues (Lys9 and Lys26), reaction conditions used mainly produced a mono-biotinylated Ac2-26 homogeneously modified at Lys26. Reaction yield was about 70% and the mono-biotinylated Ac2-26 was purified by HPLC, using a Luna C18 (1×150 mm) column and gradient from 5% to 35% of CH_3_CN in 20 min.

To prepare C2C12 membrane protein extracts, cell organelles were separated by ultra-centrifugation. Cells ruptured by sonication were centrifuged at 300 × *g* for 5 min to remove coarse debris and intact cells and the supernatant were removed and resuspended in 1 ml lysis buffer (Tris HCl 20 mM, pH 7,4; Sucrose 250 mM; DTT 1 mM; Protease inhibitors; EDTA 1 mM; H_2_O). An initial centrifugation at 13,000 × *g* separated nuclei, mitochondria and other dense material. The supernatant from this step was then resuspended in 0.5 ml lysis buffer and centrifuged for 1 h at 100,000 × *g*. The resulting pellet was resuspended in lysis buffer containing 0.1% Triton-X 100 and incubated on orbital shaker over night. The sample was then centrifuged for 30 min at 300×g and the supernatants (membrane soluble fraction) were analyzed to determine the total protein concentration using the BioRad Protein Assay Method (Bio-Rad Laboratories) according to the manufacturer's instructions.

500 µg of membrane protein extract were incubated with 50 µg of biotinylated Ac2-26 or with 12 nmol of PEG4-biotin for 1.5 h at room temperature; successively each mixture was incubated streptavidin resin for 3 h at 4°C with continuous shaking on a rotator tube holder. The beads were then washed three times with lysis buffer and then three times with PBS 1X, 0.1% Igepal. The elution of interacting proteins was performed with 50 µl of Leammli buffer (60 mM Tris HCl pH 6.8, 2% sodium dodecylsulfate, 10% glycerol, 0.01% blue bromophenol, 5% β-mercaptoethanol). Eluted samples were loaded on a mono-dimensional 12% SDS-PAGE, and separated proteins were stained with Brilliant Blue G-Colloidal (Sigma Aldrich). To perform in gel trypsin digestions, coomassie-stained protein bands were excised from the polyacrylamide gel, reduced, alkylated using iodoacetamide, and digested by trypsin. The resulting fragments were extracted and analyzed by LC/MS/MS using a Q-TOF premier instrument (Waters, Milford, USA) equipped by a nano-ESI source coupled with a nano-Aquity capillary UPLC (Waters): peptide separation was performed on a capillary BEH C18 column (0.075 mm × 100 mm, 1.7 µm, Waters) using aqueous 0.1% formic acid (A) and CH_3_CN containing 0.1% formic acid (B) as mobile phases. Peptides were eluted by means of linear gradient from 5% to 50% of B in 45 min and a 300 nl min flow rate. Capillary ion source voltage was set at 2.5 kV, cone voltage at 35 V, and extractor voltage at 3 V. Peptide fragmentation was achieved using argon as collision gas and a collision cell energy of 25 eV. Mass spectra were acquired in a *m*/*z* range from 400 to 1800, and MS/MS spectra in a 25–2000 range. Mass and MS/MS spectra calibration was performed using a mixture of angiotensin and insulin as external standard and [Glu]-Fibrinopeptide B human as lock mass standard. MS and MS/MS data were used by Mascot (Matrix Science) and Protein Prospector 5.1.8 basic (UCSF) to interrogate the Swiss Prot non-redundant protein database. Settings were as follows: mass accuracy window for parent ion, 50 ppm; mass accuracy window for fragment ions, 200 millimass units; fixed modification, carbamidomethylation of cysteines; variable modifications, oxidation of methionine.

## Results

### Extracellular Expression of ANXA1 during Skeletal Muscle Differentiation

Our previous studies [27] indicate that the administration of an ANXA1 neutralizing antibody in C2C12 cells caused reduction in myogenic differentiation.

In several systems, ANXA1 actions are exerted extracellularly via membrane-bound receptors on adjacent sites after translocation of protein from the cytoplasm onto the cell surface. Accordingly, we examined the translocation of ANXA1 onto cell membrane during C2C12 myogenic differentiation.

**Figure 1:**
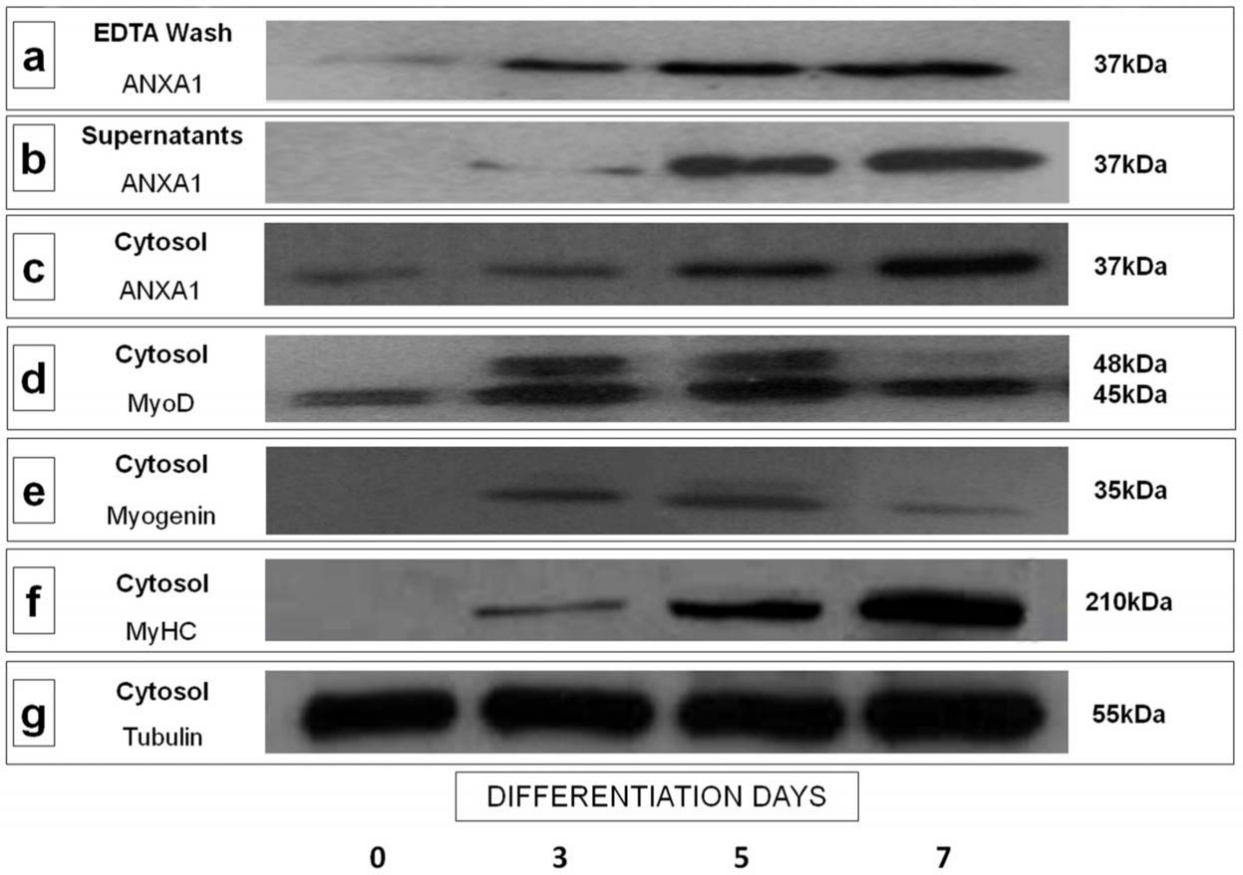
Cell surface translocation and secretion of ANXA1 during myogenic differentiation in C2C12 cells. Cell surface (a) and extracellular (b) ANXA1 from C2C12 cells in GM (0 differentiation day) and after exposure for the indicated times (3, 5, and 7 differentiation days) to DM was analyzed by Western blot with anti-ANXA1 (a, b) antibody. Total cell protein extracts were analyzed by Western blot with anti-ANXA1 (c) and with anti-MyoD (d), anti-Myogenin (e), and anti-MyHC (f) antibodies to assess myogenic differentiation rate. The protein bands were normalized on tubulin levels (g). The data are representative of 5 experiments with similar results.

Extracellular and cytosolic ANXA1 during C2C12 differentiation process was detected by Western blot analysis (Fig. 1, a-c) together with the differentiation markers MyoD (Fig. 1, d), Myogenin (Fig. 1, e) and MyHC (Fig. 1, f). Protein normalization was performed on tubulin levels (Fig. 1, g).

Our results show that resting C2C12 contains a small proportion of membrane pool ANXA1 (Fig. 1, a). This arrangement changes at 3 days of differentiation when the ANXA1 membrane pool increases remaining steady until terminal differentiation (Fig. 1, a). At the same experimental point (3 days), the protein starts to be massively secreted outside the cells (Fig. 1, b). The analysis of ANXA1 cytosolic expression (Fig. 1, c) showed that during C2C12 myogenic differentiation occurs an overall increase of the synthesis of the protein, confirming our previous data [27].

### C2C12 Cells Express Fpr-rs1 and Fpr-rs2 that are Activated by Ac2-26 Peptide

The regulatory action on cell surface by extracellular ANXA1 could be mediated by signaling through FPRs.

**Figure 2:**
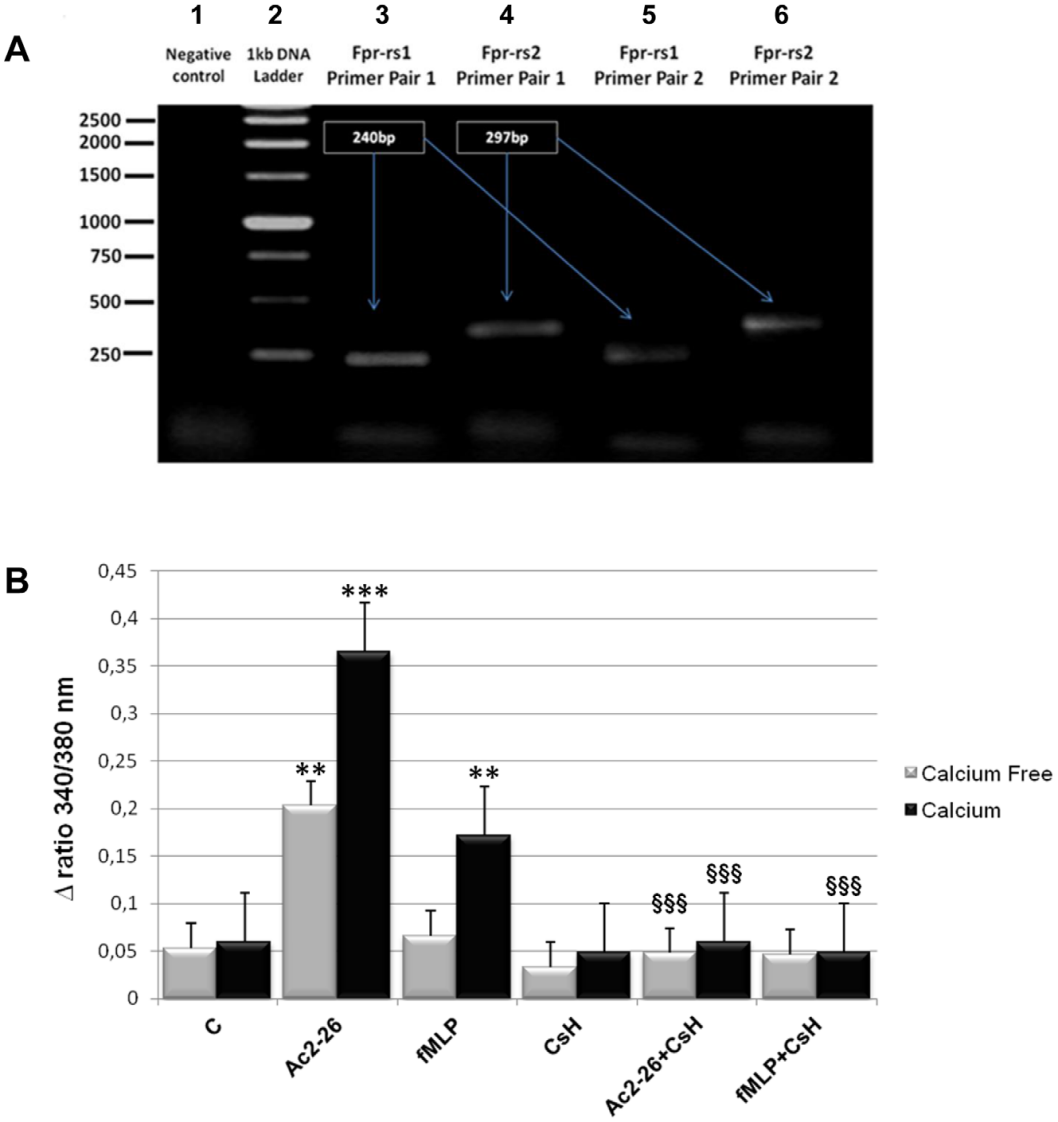
FPR detection and effects of Ac2-26, fMLP and CsH on the FPR-induced rise in intracellular Ca^2+^. (A) Qualitative PCR products for full-length Fpr-rs1 and Fpr-rs2 genes with only cDNA isolated from C2C12 cells. Product electrophoresis was performed on 1% agarose gel stained with ethidium bromide. Lane 1: negative control. Lane 2∶1 kb DNA ladder. Lane 3: primer pairs 1 for Fpr-rs1 amplicon, 240 bp. Lane 4: primer pairs 1 for Fpr-rs2 amplicon, 297 bp. Lane 5: primer pairs 2 for Fpr-rs1 amplicon, 240 bp. Lane 6: primer pairs 2 for Fpr-rs2 amplicon, 297 bp. **(B)** C2C12 were treated as described in Materials and Methods. The histogram shows the fluorescence ratio calculated as F340/F380 nm in the presence or in the absence of extracellular Ca^2+^.Control represents unstimulated cells. Data are means ± SEM (n = 3). *** <0.001, ** <0.01 vs corresponding controls; §§§ <0.001 vs Ac 2-26 or fMLP.

On the basis of the existing evidences we examined the expression of the two most important FPR superfamily receptors in C2C12 myoblast cell line by qualitative PCR. Our results show that C2C12 cells express Fpr-rs1 and Fpr-rs2 (Fig. 2A).

Although the signal transduction pathway of FPRs is partially unclear, previous studies on these and other Gi-coupled receptors have suggested that their activation often leads to the release of Ca^2+^ from intracellular stores and to the subsequent influx across the plasma membrane, which is for example essential to neutrophil chemotaxis [30].

Accordingly, we performed the measurement of the intracellular calcium mobilization following cell stimulation by known agonists/antagonists of FPRs and by the ANXA1-derived NH_2_-terminal peptide Ac2-26 (100 nM). Our results show that the well known FPR agonist fMLP (50 nM) induces appreciable calcium mobilization only in high calcium conditions (Fig. 2B) whereas Ac2-26 peptide is able to induce calcium mobilization in both high and calcium-free conditions (Fig. 2B). This pattern of calcium mobilization is not observed in cells treated with the two peptides and the FPR antagonist CsH (500 nM).

### ANXA1-derived Peptide Ac2-26 Induces C2C12 Cell Migration

To determine if ANXA1 influences myoblast cell migration acting through FPR receptors, we performed a wound-healing assay on C2C12 monolayer cell line in the presence of the FPR agonist fMLP, the FPR antagonist CsH, and the ANXA1-derived NH_2_-terminal peptide Ac2-26.

The confluent cultures were scraped to create a wound and cell migration was monitored by time-lapse video-microscopy at the site of the wound. We measured the migration distances of selected cells at different time points as previously described in Materials and Methods.

**Figure 3:**
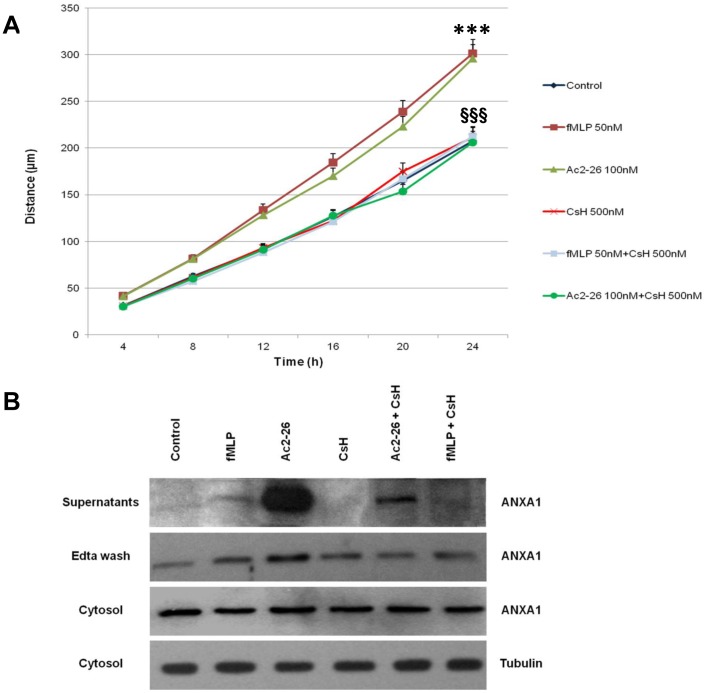
Cell surface translocation and secretion of ANXA1 after Ac2-26 treatment in Wound-healing migration assay of C2C12 cells. (**A**) Results for control, fMLP, Ac2-26, CsH, fMLP + CsH and Ac2-26+ CsH are reported as means of three experiments, measuring individual cell migrations at different times. Bars represent standard errors. (**B**) Cytosolic, cell surface and extracellular ANXA1 from C2C12 cells after exposure for the indicated time (16 h) to fMLP, Ac2-26, CsH, Ac2-26+ CsH and fMLP + CsH were analyzed by Western blot with anti-ANXA1 and anti-tubulin antibodies. The data are representative of 5 experiments with similar results. *** <0.001 vs control; §§§ <0.001 vs Ac 2-26 or fMLP.

Results in figure 3 A show a progressive increase in migration speed of cells treated with ANXA1 NH_2_-terminal peptide Ac2-26 (100 nM) or fMLP (50 nM) compared to control cells at different times after scraping (4, 8, 12, 16, 20, and 24 h). The stimulation of cell migration by either Ac2-26 or fMLP was inhibited by the FPR antagonist CsH (500 nM) (Fig. 3A).

Moreover, cell protein extracts from 16 h C2C12 wounded cells show that cell treatment with peptide Ac2-26 (100 nM) caused significant changes in ANXA1 intracellular location since after Ac2-26 strongly increases ANXA1 membrane pool (Fig. 3B). Changes in ANXA1 concentrations in cell supernatants were also detected after 16 h of Ac2-26 treatment implying the completion of the ANXA1 externalization process after its exposure on the plasma membrane.

This expression pattern is not observed in all the other experimental points including when the FPR1 high affinity agonist fMLP is used, suggesting a selective effect in ANXA1 mobilization by Ac2-26 in C2C12 cells (Fig. 3B). Interestingly, in all experimental points is not observed a reduction in ANXA1 cytosolic content (Fig. 3B), probably due to a partial replenishment of the cytosolic pool of the protein at this time point.

### ANXA1-derived Peptide Ac2-26 Potentially Interacts with Fpr-rs 2 in C2C12 Cell Line

To identify which of the eight FPR isoforms expressed in mice interacts with ANXA1-derived peptide Ac2-26, chemical proteomics experiments were performed [31], using peptide Ac2-26 as a probe. Preliminarily the peptide was biotinylated at Lys26 in order to allow the affinity chromatography purification of the possible complexes it would form with proteins. A membrane protein extract of C2C12 cell line was then incubated with biotinylated Ac2-26; the same incubation was also performed using biotin to obtain a control sample, needed to distinguish between specifically bound components and background contaminants. Both the samples were purified by affinity chromatography on a streptavidin resin and the resulting protein mixtures were resolved by SDS-PAGE; the gel line was cut in 13 pieces, digested with trypsin, and analyzed by mass spectrometry through nanoflow reversed-phase HPLC MS/MS. Doubly and triply charged peptide species were fragmented, and all the MS/MS spectra were evaluated by a Mascot database search.

The list of the identified proteins was compared with that in the control experiment. This analysis led to the identification of three potential partners (Table 1) of the ANXA1-derived peptide Ac2-26. All those proteins were recognized in three different experiments, by at least 9 non-redundant peptides and minimum sequence coverage of 25%. Our results show that in C2C12 myoblasts Ac2-26 potentially interacts with Fpr-rs2 receptor with sequence coverage of 25% by using 9 different peptides (Table 1).

**Table 1:** Proteins identified as possible Ac2-26 partners by chemical proteomics.

Swiss Prot code	Identified protein	Sequence coverage (%)	Peptides
ACTS_MOUSE	Actin, Alpha scheletal muscle	32	14
FPR2_MOUSE	Formyl Peptide Receptor 2	25	9
ANXA1_MOUSE	Annexin A1	28	10

### Ca^2+^-dependent Cellular Relocation of ANXA1 in C2C12 Myoblast Cell Line

Our previous data showed that ANXA1 accumulates at the protruding ends of active migrating cells and interacts with F-actin [27]. This could be due to the Ca^2+^-sensitivity of the protein and could reflect a role for ANXA1 as a highly versatile component in the signaling chains triggered by the proper calcium perturbation that takes place during active migration and differentiation as well as following FPR activation.

On the basis of these evidences, we suppose the existence of a positive loop by which ANXA1 is produced within, and exported outside the cells, where it stimulates FPRs, inducing intracellular calcium release and ANXA1 accumulation at the protruding ends of active migrating cells possibly interacting in a calcium-dependent manner with F-actin.

In order to deep into this aspect, we analyzed the effects of a strong intracellular calcium perturbation on ANXA1 mobilization in skeletal muscle cells.

**Figure 4:**
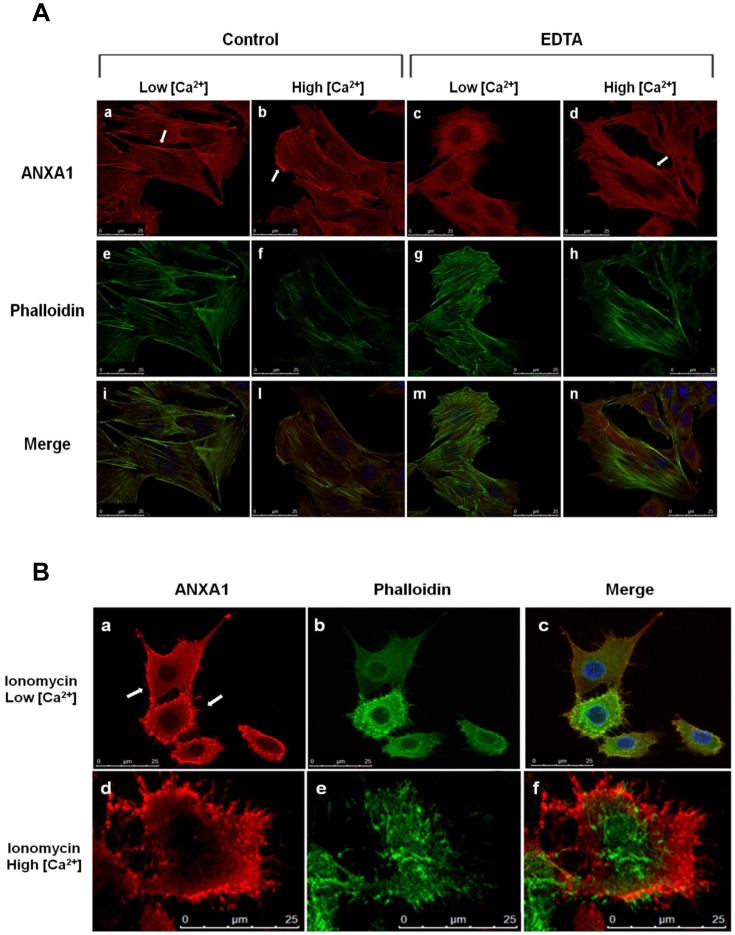
ANXA1 cellular relocation following Ca^2+^ challenge. (A) Cultured murine C2C12 myoblasts fixed and labeled with fluorescent antibody against ANXA1 and with FITC-conjugated Phalloidin in Ca^2+^-free conditions (a, c, e, g) and in a medium containing 2 mM Ca^2+^ (b, d, f, h). **(B)** An increase of intracellular Ca^2+^ levels leads to ANXA1 relocation to the plasma membrane (a); at high Ca^2+^ concentrations actin filaments (green) are significantly damaged by high intracellular increase of Ca^2+^ level (e): in this condition ANXA1(red) is strikingly shifted to the plasma membrane (d). Bar = 25 µm.

In Ca^2+^-free conditions immunolabeling of C2C12 cells, which possess a well-developed stress fiber system, ANXA1 has an obvious filamentous organization as well as a diffusely distribution throughout the cytoplasm (Fig. 4A, panels a, e, i). An increase of intracellular [Ca^2+^] (achieved by adding 2 mM Ca^2+^ to the culture medium) leads the protein to mainly localize at the leading edges of C2C12 cells (Fig. 4A, panels b, f, l).

At low intracellular [Ca^2+^] (obtained by incubating cells in medium containing 2 mM EDTA without Ca^2+^), immunolabeling revealed ANXA1 to be diffusely distributed throughout the cytoplasm, with no obvious filamentous organization (Fig. 4A, panels c, g, m) that is partially restored when 2 mM Ca^2+^ is added (Fig. 4A, panels d, h, n).

Increased intracellular levels of Ca^2+^ (achieved by incubating cells with the Ca^2+^ ionophore Ionomycin) lead to ANXA1 translocation to the plasma membrane (Fig. 4B, panels a-c): this redistribution of the protein is strongly visible at very high intracellular levels of Ca^2+^ when the stress-fiber system has been hard damaged by the abrupt increase of the Ca^2+^ arising from the treatment with ionophore Ionomycin and 2 mM Ca^2+^ (Fig. 4B, panels d-f).

## Discussion

ANXA1 is involved in a wide range of functions both inside and outside cells such as membrane aggregation, inflammation, phagocytosis, apoptosis, proliferation, and differentiation. Cellular ANXA1 knockdowns and mouse knockout models have revealed processes that are affected by the loss of ANXA1. As expected, these events are often linked to Ca^2+^ signaling and membrane functions, although in some cases extracellular functions have been revealed, for example, in the regulation of inflammatory reactions and fibrinolytic homeostasis.

As mentioned above, our previous studies [27] indicate that ANXA1 could be a novel determinant for tissue repair, at least in the muscle, playing a role in stem cell (SCs in the muscle) migration and differentiation.

It is well known that the establishment of a set of environmental factors, namely soluble factors, regulates the activation of myogenic factors and the progression of myoblast differentiation through a complex interplay of signaling pathways, including the activation of calcineurin and NFAT, Rho/Rho kinase, PI-3-kinase and p38 MAPK cascades [32]–[36]. Since ANXA1 has long been known to occur extracellularly under conditions of inflammation, and it shows potent anti-inflammatory activities [7], [37]–[38], mainly interacting with specific receptors on leukocytes [39], we investigated ANXA1 membrane translocation and secretion during C2C12 active migration, one of the first steps in the processes of skeletal muscle maintenance and regeneration once the SCs are committed to differentiate.

Our results show that resting C2C12 contain a small amount of ANXA1 in the membrane pool and that this arrangement changes at 3 days of differentiation when the ANXA1 membrane pool increases remaining steady until terminal differentiation. Interestingly, at 3 days of differentiation the protein also starts to be secreted outside the cells. Analysis of cytosolic expression of ANXA1 protein confirms our previous data [27] that is the cellular content of the protein increases during C2C12 myogenic differentiation.

In several systems, ANXA1 actions are exerted extracellularly via membrane-bound receptors on adjacent sites after translocation of protein from the cytoplasm onto the cell surface. The ANXA1 receptors, at least on leukocytes, have been identified as members of the FPR family [40].

On the basis of the existing evidences we examined by PCR the expression of the FPR receptors in C2C12 myoblast cell line and we found that C2C12 cells express Fpr-rs1 and Fpr-rs2 isoforms.

Ligands bound to the G-coupled receptors FPRs trigger a number of signaling systems. Activation of PLCβ by Gβγ results in hydrolysis of phosphatidylinositol 4,5-bisphosphate (PIP2), generating DAG, which activates PKC isoforms, and inositol-1,4,5-trisphosphate (IP3), which releases Ca^2+^ from intracellular stores. The release of Ca^2+^ from internal stores induces the opening of the store-operated Ca^2+^ channel in the plasma membrane followed by a sustained influx of Ca^2+^ [41].

Our results show that ANXA1-derived NH_2_-terminal peptide Ac2-26 is able to induce FPR activation and intracellular calcium increase in C2C12 myoblasts: this effect on Ca^2+^ mobilization might be mainly from intracellular stores since extracellular Ca^2+^ is not required. This finding is supported by initial proteomics experiments that indicate that the Ac2-26 peptide interacts with FPR receptors, confirming what is known about ANXA1 ligands. Moreover, data obtained by this approach suggested a selective recognition of the ANXA1 N-terminus by Fpr-rs2. The identification of ANXA1 in the same analysis could possibly be due to the presence of stable Fpr-rs2/ANXA1 complexes in the membrane protein extracts from C2C12 myoblast cell line. Further experiments are necessary to better address this point.

A wound-healing assay on C2C12 monolayer cell line in the presence of the well known FPR agonist fMLP, of the FPR antagonist CsH and in the presence of the ANXA1-derived NH_2_-terminal peptide Ac2-26 show that ANXA1 influences myoblast cell migration acting through FPR receptors. In fact, our data show a progressive increase in migration speed of cells treated with ANXA1 NH_2_-terminal peptide Ac2-26 and fMLP compared to control cells at different times after scraping. This increase in migration speed was inhibited by FPR antagonist CsH.

Moreover, cell protein extracts from 16 h C2C12 wounded cells show that cell treatment with peptide Ac2-26 strongly increased ANXA1 membrane pool. Changes in ANXA1 concentrations in cell supernatants were also detected after 16 h of Ac2-26 treatment implying the completion of the ANXA1 externalization process after its exposure on the plasma membrane.

In our model [42], extracellular ANXA1 may lead a feedback loop on its function and may modulate signal transduction in a cell-activating way stimulating the migration of both SCs and myoblasts through activation of FPRs: ANXA1 is produced within and exported outside the cells, where it stimulates FPRs, inducing intracellular calcium release, PLCβ and PKC activation and F-actin polymerization. This feedback loop may be strengthened throughout a severe muscle injury in which damaged skeletal muscle cells could represent a conceivable early source for extracellular ANXA1 that could exert its effects in a paracrine manner on the neighbouring cells.

FPR ligation has been also shown to signal through the small G protein Cdc42 to activate Rac- and ARP2/3-dependent pathways leading to actin nucleation [43] and stress fiber formation.

Apart from maintaining cell shape and coordinating cell movement, cytoskeletal actin may also participate in the regulation of cell differentiation and skeletal myogenesis, representing a nodal point in the signal transduction leading to muscle formation [44]–[49].

Indeed, there is evidence that Rho-dependent regulation of muscle development is mediated by its ability to induce cytoskeletal reorganization, since either the inhibition of Rho function with C3 toxin or disruption of actin filament with Cytochalasin D are equally effective in blocking myoblast differentiation [50].

It was also shown that cytoskeleton may have a functional role in the transduction of differentiation signals in C2C12 murine myoblasts, where the formation of stress fibers in response to sphingosine 1-phosphate, for example, is able to transmit a mechanical tension to the plasma membrane and, in turn, stimulate stretch-activated channels (SACs) and Ca^2+^ influx [51]. These data couple with the known role played by extracellular Ca^2+^ on muscle differentiation [52] and suggests that actin cytoskeletal reorganization and SAC opening may represent critical events in the differentiative processes of myogenic cells.

In parallel to act extracellularly, ANXA1 protein could take part in the process of cytoskeleton reorganization following the calcium perturbation that take place during myogenic cell migration and differentiation or next FPR activation.

In this regard, we show that in Ca^2+^-free conditions immunolabeling of C2C12 cells, which possess a well-developed stress fiber system, ANXA1 has an obvious filamentous organization as well as a diffusely distribution throughout the cytoplasm whereas an increase of Ca^2+^ concentration, as occurs during muscle cell migration and differentiation [52], leads the protein to mainly localize at the leading edges of C2C12 cells. High levels of Ca^2+^ in the culture medium lead to ANXA1 translocation to the plasma membrane: this redistribution of the protein is strongly visible at very high concentrations of Ca^2+^ when the stress-fiber system and plasma membrane have been hard damaged by the abrupt increase of the ion as could happen in a muscle injury scenario. The observed subcellular relocation of ANXA1 protein in C2C12 myoblasts in response to the changes of Ca^2+^ concentration should be related to what was previously described by Lennon et al. that showed an interesting association between dysferlin and ANXA1 in a Ca^2+^ and membrane injury-dependent manner assuming that [53].

Consistently, in the area of gut pathology, properties similar to those we have described in this work for exogenous and endogenous ANXA1 in epithelial cell differentiation and motility were shown [54], well complemented by the observation that ANXA1-null mice delay their repair of the gut upon application of a model of colitis [55]. It is highly plausible that our findings would lead to a novel approach to the promotion of the repair of an injured skeletal muscle and to therapeutic implications with respect to the development of ANXA1 mimetics.

## References

[1] Marfella R, Sasso FC, Cacciapuoti F, Portoghese M, Rizzo MR, et al (2012) Tight glycemic control may increase regenerative potential of myocardium during acute infarction. J Clin Endocrinol Metab 97: 933–942. 22170713 10.1210/jc.2011-2037

[2] Mauro A (1961) Satellite cell of skeletal muscle fibers. J Biophys Biochem Cytol 9: 493–495. 13768451 10.1083/jcb.9.2.493 PMC2225012

[3] Hawke TJ, Garry DJ (2001) Myogenic satellite cells: Physiology to molecular biology. J Appl Physiol 91: 534–551. 11457764 10.1152/jappl.2001.91.2.534

[4] Ervasti JM (2003) Costameres: The Achille’s heel of Herculean muscle. J Biol Chem 278: 13591–13594. 12556452 10.1074/jbc.R200021200

[5] Siegel AL, Atchison K, Fisher KE, Davis GE, Cornelison DD (2009) 3D timelapse analysis of muscle satellite cell motility. Stem Cells 27: 2527–2538. 19609936 10.1002/stem.178 PMC2798070

[6] Lim LH, Pervaiz S (2007) Annexin 1: The new face of an old molecule. FASEB J 21: 968–975. 17215481 10.1096/fj.06-7464rev

[7] Perretti M, D’Acquisto F (2009) AnnexinA1 and glucocorticoids as effectors of the resolution of inflammation. Nat Rev Immunol 9: 62–70. 19104500 10.1038/nri2470

[8] Gil CD, La M, Perretti M, Oliani SM (2006) Interaction of human neutrophils with endothelial cells regulates the expression of endogenous proteins annexin 1, galectin-1 and galectin-3. Cell Biol Int 30: 338–344. 16530434 10.1016/j.cellbi.2005.12.010

[9] Williams SL, Milne IR, Bagley CJ, Gamble JR, Vadas MA, et al (2010) A proinflammatory role for proteolytically cleaved annexin A1 in neutrophil transendothelial migration. J Immunol 185: 3057–3063. 20679535 10.4049/jimmunol.1000119

[10] Lange C, Starrett DJ, Goetsch J, Gerke V, Rescher U (2007) Transcriptional profiling of human monocytes reveals complex changes in the expression pattern of inflammation-related genes in response to the annexin A1-derived peptide Ac1–25. J Leukoc Biol 82: 1592–1604. 17855500 10.1189/jlb.0307158

[11] Monastyrskaya K, Babiychuk EB, Draeger A (2009) The annexins: Spatial and temporal coordination of signaling events during cellular stress. Cell Mol Life Sci 66: 2623–2642. 19381436 10.1007/s00018-009-0027-1 PMC11115530

[12] Morello S, Petrella A, Festa M, Popolo A, Monaco M, et al (2008) Cl-IB-MECA inhibits human thyroid cancer cell proliferation independently of A3 adenosine receptor activation. Cancer Biol Ther 7: 278–284. 18059189 10.4161/cbt.7.2.5301

[13] Scannell M, Maderna P (2006) Lipoxins and annexin-1: Resolution of inflammation and regulation of phagocytosis of apoptotic cells. Scientific World J 6: 1555–1573. 10.1100/tsw.2006.259 PMC5917148 17160341

[14] Huo X, Zhang JW (2005) Annexin1 regulates the erythroid differentiation through ERK signaling pathway. Biochem Biophys Res Commun 331: 1346–1352. 15883023 10.1016/j.bbrc.2005.04.049

[15] Hullin F, Raynal P, Ragab-Thomas JM, Fauvel J, Chap H (1989) Effect of dexamethasone on prostaglandin synthesis and on lipocortin status in human endothelial cells. Inhibition of prostaglandin I2 synthesis occurring without alteration of arachidonic acid liberation and of lipocortin synthesis. J Biol Chem 264: 3506–3513. 2521636

[16] Ambrose MP, Hunninghake GW (1990) Corticosteroids increase lipocortin I in alveolar epithelial cells. Am J Respir Cell Mol Biol 3: 349–353. 2144977 10.1165/ajrcmb/3.4.349

[17] Croxtall JD, Choudhury Q, Newman S, Flower RJ (1996) Lipocortin 1 and the control of cPLA2 activity in A549 cells. Glucocorticoids block EGF stimulation of cPLA2 phosphorylation. Biochem Pharmacol 52: 351–356. 8694860 10.1016/0006-2952(95)02442-5

[18] Perretti M, Croxtall JD, Wheller SK, Goulding NJ, Hannon R, et al (1996) Mobilizing lipocortin 1 in adherent human leukocytes downregulates their transmigration. Nat Med 2: 1259–1262. 8898757 10.1038/nm1196-1259

[19] Rhee HJ, Kim GY, Huh JW, Kim SW, Na DS (2000) Annexin I is a stress protein induced by heat, oxidative stress and a sulfhydryl-reactive agent. Eur J Biochem 267: 3220–3225. 10824106 10.1046/j.1432-1327.2000.01345.x

[20] Sampey AV, Hutchinson P, Morand EF (2000) Annexin I surface binding sites and their regulation on human fibroblast-like synoviocytes. Arthritis Rheum 43: 2537–2542. 11083278 10.1002/1529-0131(200011)43:11<2537::AID-ANR22>3.0.CO;2-M

[21] Cheng TY, Wu MS, Lin JT, Lin MT, Shun CT, et al.. (2012) Annexin A1 is associated with gastric cancer survival and promotes gastric cancer cell invasiveness through the formyl peptide receptor/extracellular signal-regulated kinase/integrin beta-1-binding protein 1 pathway. Cancer doi: 10.1002/cncr.27565. 10.1002/cncr.27565 22736399

[22] Perretti M, D'Acquisto F (2009) Annexin A1 and glucocorticoids as effectors of the resolution of inflammation. Nat Rev Immunol 9: 62–70. 19104500 10.1038/nri2470

[23] Dalli J, Montero-Melendez T, McArthur S, Perretti M (2012) Annexin A1 N-terminal derived Peptide ac2–26 exerts chemokinetic effects on human neutrophils. Front Pharmacol 3: 28. 22403546 10.3389/fphar.2012.00028 PMC3288723

[24] Ye RD, Boulay F, Wang JM, Dahlgren C, Gerard C, et al (2009) International Union of Basic and Clinical Pharmacology. LXXIII. Nomenclature for the formyl peptide receptor (FPR) family. Pharmacol Rev 61: 119–161. 19498085 10.1124/pr.109.001578 PMC2745437

[25] Huang J, Chen K, Chen J, Gong W, Dunlop NM, et al (2010) The G-protein-coupled formylpeptide receptor FPR confers a more invasive phenotype on human glioblastoma cells. Br J Cancer 102: 1052–1060. 20197768 10.1038/sj.bjc.6605591 PMC2844039

[26] Huang J, Chen K, Gong W, Dunlop NM, Wang JM (2008) G-protein coupled chemoattractant receptors and cancer. Front Biosci 13: 3352–3363. 18508437 10.2741/2930 PMC7422331

[27] Bizzarro V, Fontanella B, Franceschelli S, Pirozzi M, Christian H, et al (2010) Role of Annexin A1 in mouse myoblast cell differentiation. J Cell Physiol 224: 757–765. 20578244 10.1002/jcp.22178

[28] Sur P, Sribnick EA, Wingrave JM, Nowak MW, Ray SK, et al (2003) Estrogen attenuates oxidative stress-induced apoptosis in C6 glial cells. Brain Res. 971: 178–88. 10.1016/s0006-8993(03)02349-7 12706234

[29] Grynkiewicz G, Poenie M, Tsien RY (1985) A new generation of Ca^2+^ indicators with greatly improved fluorescence properties. J Biol Chem 260: 3440–3450. 3838314

[30] Dufton N, Perretti M (2010) Therapeutic anti-inflammatory potential of formyl-peptide receptor agonists. Pharmacol Ther 127: 175–188. 20546777 10.1016/j.pharmthera.2010.04.010

[31] Rix U, Superti-Furga G (2009) Target profiling of small molecules by chemical proteomics. Nat Chem Biol 9: 616–624. 10.1038/nchembio.216 19690537

[32] Wei L, Zhou W, Wang L, Schwartz RJ (2000) beta(1)-integrin and PI 3-kinase regulate RhoA-dependent activation of skeletal alpha-actin promoter in myoblasts. Am J Physiol Heart Circ Physiol 278: 1736–1743. 10.1152/ajpheart.2000.278.6.H1736 10843867

[33] Cabane C, Englaro W, Yeow K, Ragno M, Dérijard B, et al (2003) Regulation of C2C12 myogenic terminal differentiation by MKK3/p38alpha pathway. Am J Physiol Cell Physiol 284: 658–666. 10.1152/ajpcell.00078.2002 12444016

[34] Khurana A, Dey CS (2003) p38 MAPK interacts with actin and modulates filament assembly during skeletal muscle differentiation. Differentiation 71: 42–50. 12558602 10.1046/j.1432-0436.2003.700604.x

[35] Foulstone EJ, Huser C, Crown AL, Holly JM, Stewart CE (2004) Differential signalling mechanisms predisposing primary human skeletal muscle cells to altered proliferation and differentiation: roles of IGF-I and TNF alpha. Exp Cell Res 294: 223–235. 14980516 10.1016/j.yexcr.2003.10.034

[36] Schulz RA, Yutzey KE (2004) Calcineurin signaling and NFAT activation in cardiovascular and skeletal muscle development. Dev Biol 266: 1–16. 14729474 10.1016/j.ydbio.2003.10.008

[37] D’Acquisto F, Perretti M, Flower RJ (2008) Annexin-A1: A pivotal regulator of the innate and adaptive immune systems. Br J Pharmacol 155: 152–169. 18641677 10.1038/bjp.2008.252 PMC2538690

[38] Perretti M, Dalli J (2009) Exploiting the Annexin A1 pathway for the development of novel anti-inflammatory therapeutics. Br J Pharmacol 158: 936–946. 19845684 10.1111/j.1476-5381.2009.00483.x PMC2785517

[39] Gerke V, Moss SE (2002) Annexins: From structure to function. Physiol Rev 82: 331–371. 11917092 10.1152/physrev.00030.2001

[40] Walther A, Riehemann K, Gerke V (2000) A novel ligand of the formyl peptide receptor: Annexin 1 regulates neutrophil extravasation by interacting with the FPR. Mol Cell 5: 831–840. 10882119 10.1016/s1097-2765(00)80323-8

[41] Ye RD, Boulay F, Wang JM, Dahlgren C, Gerard C, et al (2009) International Union of Basic and Clinical Pharmacology. LXXIII. Nomenclature for the formyl peptide receptor (FPR) family. Pharmacol Rev 61: 119–161. 19498085 10.1124/pr.109.001578 PMC2745437

[42] Bizzarro V, Petrella A, Parente L (2012) Annexin A1: Novel roles in skeletal muscle biology. J Cell Physiol 227: 3007–3015. 22213240 10.1002/jcp.24032

[43] VanCompernolle SE, Clark KL, Rummel KA, Todd SC (2003) Expression and function of formyl peptide receptors on human fibroblast cells. J Immunol 171: 2050–2056. 12902510 10.4049/jimmunol.171.4.2050

[44] Ballestrem C, Wehrle-Haller B, Imhof BA (1998) Actin dynamics in living mammalian cells. J Cell Sci 111: 1649–1658. 9601095 10.1242/jcs.111.12.1649

[45] Huang S, Ingber DE (2000) Shape-dependent control of cell growth, differentiation, and apoptosis: switching between attractors in cell regulatory networks. Exp Cell Res 261: 91–103. 11082279 10.1006/excr.2000.5044

[46] Ingber DE (2003) Mechanobiology and diseases of mechanotransduction. Ann Med 35: 564–577. 14708967 10.1080/07853890310016333

[47] Dhawan J, Helfman DM (2004) Modulation of acto-myosin contractility in skeletal muscle myoblasts uncouples growth arrest from differentiation. J Cell Sci 117: 3735–3748. 15252113 10.1242/jcs.01197

[48] McBeath R, Pirone DM, Nelson CM, Bhadriraju K, Chen CS (2004) Cell shape, cytoskeletal tension, and RhoA regulate stem cell lineage commitment. Dev Cell 6: 483–495. 15068789 10.1016/s1534-5807(04)00075-9

[49] Komati H, Naro F, Mebarek S, De Arcangelis V, Adamo S, et al (2005) Phospholipase D is involved in myogenic differentiation through remodeling of actin cytoskeleton. Mol Biol Cell 16: 1232–1244. 15616193 10.1091/mbc.E04-06-0459 PMC551488

[50] Lee KH, Lee SH, Kim D, Rhee S, Kim C, et al (1999) Promotion of skeletal muscle differentiation by K252a with tyrosine phosphorylation of focal adhesion: a possible involvement of small GTPaseRho. Exp Cell Res 252: 401–415. 10527630 10.1006/excr.1999.4648

[51] Formigli L, Meacci E, Sassoli C, Chellini F, Giannini R, et al (2005) Sphingosine 1-phosphate induces cytoskeletal reorganization in C2C12 myoblasts: physiological relevance for stress fibres in the modulation of ion current through stretch-activated channels. J Cell Sci 118: 1161–1171. 15728255 10.1242/jcs.01695

[52] De Arcangelis V, Coletti D, Canato M, Molinaro M, Adamo S, et al (2005) Hypertrophy and transcriptional regulation induced in myogenic cell line L6-C5 by an increase of extracellular calcium. J Cell Physiol 202: 787–795. 15389566 10.1002/jcp.20174

[53] Lennon NJ, Kho A, Bacskai BJ, Perlmutter SL, Hyman BT, et al (2003) Dysferlin interacts with annexins A1 and A2 and mediates sarcolemmal wound-healing. J Biol Chem 278: 50466–50473. 14506282 10.1074/jbc.M307247200

[54] Babbin BA, Lee WY, Parkos CA, Winfree LM, Akyildiz A, et al (2006) Annexin I regulates SKCO-15 cell invasion by signaling through Formyl Peptide Receptors. JBC 28: 19588–19599. 10.1074/jbc.M513025200 16675446

[55] Babbin BA, Laukoetter MG, Nava P, Koch S, Lee WY, et al (2008) AnnexinA1 regulates intestinal mucosal injury, inflammation, and repair. J Immunol 181: 5035–5044. 18802107 10.4049/jimmunol.181.7.5035 PMC2778483

